# The impact of documentation of severe acute kidney injury on mortality 

**DOI:** 10.5414/CN108072

**Published:** 2013-09-30

**Authors:** Francis Perry Wilson, Amar D. Bansal, Sravan K. Jasti, Jennie J. Lin, Michael G.S. Shashaty, Jeffrey S. Berns, Harold I Feldman, Barry D. Fuchs

**Affiliations:** 1 Department of Medicine,; 2 Department of Biostatistics and Epidemiology, and; 3 Center for Clinical Epidemiology and Biostatistics, Perelman School of Medicine at the University of Pennsylvania, Philadelphia, PA, USA

**Keywords:** acute kidney injury, documentation, mortality, cohort studies, international classification of diseases

## Abstract

Aims: Modification of the mortality risk associated with acute kidney injury (AKI) necessitates recognition of AKI when it occurs. We sought to determine whether formal documentation of AKI in the medical record, assessed by billing codes for AKI, would be associated with improved clinical outcomes. Methods: Retrospective cohort study conducted at three hospitals within a single university health system. Adults without severe underlying kidney disease who suffered in-hospital AKI as defined by a doubling of baseline creatinine (n = 5,438) were included. Those whose AKI was formally documented according to discharge billing codes were compared to those without such documentation in terms of 30-day mortality. Results: Formal documentation of AKI occurred in 2,325 patients (43%). Higher baseline creatinine, higher peak creatinine, medical admission status, and higher Sequential Organ Failure Assessment (SOFA) score were strongly associated with documentation of AKI. After adjustment for severity of disease, formal AKI documentation was associated with reduced 30-day mortality – OR 0.81 (0.68 – 0.96, p = 0.02). Patients with formal documentation were more likely to receive a nephrology consultation (31% vs. 6%, p < 0.001) and fluid boluses (64% vs. 45%, p < 0.001), and had a more rapid discontinuation of angiotensin-converting enzyme inhibitor and angiotensin-receptor blocker medications (HR 2.04, CI 1.69 – 2.46, p < 0.001). Conclusions: Formal documentation of AKI is associated with improved survival after adjustment for illness severity among patients with creatinine-defined AKI.

## Introduction 

Acute kidney injury (AKI) carries an independent risk of mortality among hospitalized patients [1, 2]. Recent studies have demonstrated increased mortality among patients with even small increases in serum creatinine concentration [3, 4]. International guidelines for the treatment of AKI focus on appropriate management of drug dosing, avoiding nephrotoxic exposures, and careful attention to fluid and electrolyte balance [5]. Early nephrologist involvement may also improve outcomes in AKI [6]. Without appropriate provider recognition of AKI, however, none of these measures can be taken, and patient outcomes may suffer. 

The Acute Kidney Injury Network (AKIN) consensus criteria enumerate creatinine and urine output metrics to define AKI [7], but a study examining chart documentation found that AKI may be appropriately documented in the medical record in as little as 16% of patients with AKI based on serum creatinine measurements [8]. Billing codes for AKI result from the formal documentation of AKI in the medical record by treating clinicians using certain key phrases. Recognition of AKI by treating clinicians may occur in the absence of this formal documentation [9, 10], but the use of billing codes as a proxy for clinical AKI recognition is common [11, 12, 13, 14]. To our knowledge, the clinical significance of formal AKI documentation among patients with laboratory-defined AKI has yet to be studied. 

We sought to determine predictors of formal documentation of AKI and the association between formal documentation and 30-day mortality within the University of Pennsylvania Health System (UPHS)-AKI cohort, a large retrospective cohort study of patients with severe in-hospital AKI defined by changes in serum creatinine. We hypothesized that the formal documentation of AKI would be associated with more severe AKI, but would associate with improved outcomes after adjustment for disease severity. We further sought to determine whether formal documentation of AKI is a valid surrogate for recognition of AKI by managing clinicians. 

## Methods 

### Patients 

This study was approved by the Institutional Review Board of the University of Pennsylvania. The UPHS-AKI cohort is a retrospective cohort that examined all adult admissions to each of three UPHS hospitals over a 7-year period. Details of the construction of the cohort have been previously published [15]. Briefly, the cohort consists of adults without severe underlying kidney disease who suffered in-hospital AKI as defined by a doubling of serum creatinine from baseline levels. Baseline creatinine was defined as the lowest serum creatinine achieved within 48 hours of hospital admission. Doubling of creatinine was chosen as an entry criterion to identify a population of patients in whom AKI was clinically obvious and highly relevant, and in whom documentation could importantly impact outcomes. Patients were excluded if they received a renal transplant during the admission or were pregnant on admission. All patients who received dialytic therapy during the hospital admission were excluded, as they would all be expected to have documentation of AKI. For patients who met inclusion criteria for more than one hospital admission, only the first admission was used. 

### Formal documentation of AKI 

We defined formal documentation of AKI as the post-hospital discharge assignment of one of five ICD-9 billing codes for AKI, as detailed in Supplementary Table 1. In our health system, ICD-9 codes are determined by chart review of clinical documentation by a trained team of professional chart abstractors. They apply these codes when any of the following phrases appear in physician documentation: ARF, AKI, acute kidney injury, acute kidney insufficiency, acute kidney failure, acute renal failure, acute tubular necrosis, ATN, papillary necrosis, and renal cortical necrosis. 

### Covariate ascertainment 

Race was determined by the hospital demographic database, which reflects patient self-report. Comorbidity data was ascertained from billing records according to the extended ICD-9 algorithm developed by Quan et al. [16] and reflect admission comorbidities. Sequential Organ Failure Assessment (SOFA) scores were calculated, but excluded the neurologic and renal components due to inadequate documentation of mental status and to allow for inclusion of serum creatinine in multivariable models [17]. Patients without recent values allowing for calculation of the hepatic (n = 2,213) or pulmonary aspects of the SOFA score (n = 14) were assigned a value of 0 in these categories, but there was no imputation required for the coagulation or cardiovascular categories. Renal consults were determined from billing records. Post-AKI interventions were determined from an electronic order entry database. Laboratory variables were collected during routine clinical care. Duration of AKI was defined as the time between development of AKI (by AKIN Stage 1 creatinine criteria) and return of creatinine to within 10% of the baseline level or discharge (whichever came first). Time from AKI onset to doubling was defined as the time between development of AKI (by AKIN Stage 1 creatinine criteria) and doubling of creatinine from baseline. Peak and nadir values of variables (max achieved creatinine, peak SOFA, etc.) reflect the peak or nadir value that occurred during the episode of AKI. 

### Validation of documentation of AKI as a surrogate for provider recognition of AKI 

**Figure 1.**
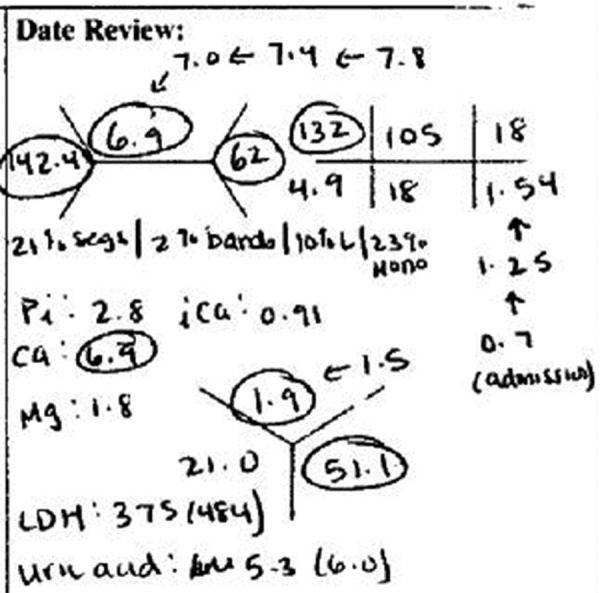
Screenshot from patient chart evidencing recognition of AKI in the absence of billable documentation (note arrows that trend the increase in creatinine from 0.7 to 1.54 mg/dl).

A subset of the overall cohort (who were admitted after 1/1/2009) was randomly selected for independent review of the medical record by two trained readers (AB, SJ), who were blinded to billing codes. Like the overall cohort, all patients in the sub-cohort had severe AKI with a doubling of serum creatinine from baseline. The readers evaluated whether a physician recognized AKI from one day before to seven days after AKI onset according to handwritten progress notes. Discrepancies were evaluated by a third blinded reviewer (FW). Phrases used by chart abstractors (as defined above) were included as evidence of recognition. In addition, other medical record notations could also be used as evidence of recognition; “rising creatinine”, “elevated creatinine” or handwritten graphical representations in the lab section of the progress note that, in the opinion of the reviewers, evidenced recognition of AKI (Figure 1). 

### Missing data and imputation 

Laboratory variables were allowed to be carried forward a maximum of 2 days to account for intermittent laboratory data. Variables with any missingness after this procedure are not included in any models. 

### Primary outcome 

The primary outcome was mortality within 30-days of AKI onset. Date of death was determined from hospital discharge records or from the Social Security Death Master File. 

### Statistical analysis 

Descriptive statistics utilized means and standard deviations to characterize normally distributed variables and medians and inter-quartile ranges to characterize non-normally distributed variables. Proportions are expressed in percentages. Student’s t-test or Wilcoxon rank sum tests were used in unadjusted comparisons of continuous variables, as appropriate. Comparisons of proportions were performed using χ^2^-testing. Adjusted associations with AKI documentation and of the relationship between AKI documentation to 30-day mortality were performed via logistic regression. Multivariable models were constructed based upon covariates found to be significant in univariable analysis and those thought to plausibly be associated with AKI documentation. Model discrimination was assessed using c-statistics. Cox proportional hazards models were used to assess time to discontinuation of angiotensin-converting enzyme (ACE) inhibitor or angiotensin receptor blocker (ARB) therapy, with time 0 defined as the onset of AKI, and outcomes censored at death, discharge, or 7 days after the onset of AKI. Schoenfeld residuals and log-log survival plots were utilized to assess for violations of the proportional hazards assumption. Model fit was assessed with the Akaike information criterion [18]. κ-statistics were used to compare inter-rater concordance in the random validation sample. 

## Results 

### Cohort characteristics 

After application of the above inclusion and exclusion criteria, 5,438 patients with severe AKI were evaluable for this study. Of these, 2,325 (43%) received a billing code for AKI. In-hospital, 30-day, and 1-year mortality rates were 17.4, 19.0, and 31.5%, respectively, for those who did not receive documentation of AKI and 25.7, 27.9, and 42% for those who were documented to have AKI during the hospitalization (p for all comparisons < 0.001). 

Table 1 describes the baseline characteristics of this cohort. Roughly 50% of the patients were male, 30% black, and 45% admitted to a surgical service. Diabetes was present in 27%; the most common comorbidity was congestive heart failure. The mean (SD) age was 61.1 (16.4) years. Median (IQR) length of stay was 14 (8 – 24) days. 

### Patient factors associated with formal AKI documentation 

In unadjusted analysis, patients in whom AKI was formally documented were more often male (58% vs. 48%, p < 0.001) and less often admitted to a surgical service (41% vs. 48%, p < 0.001). In addition, patients in whom AKI was formally documented had a longer duration of AKI and longer duration of overall hospital stay (p < 0.001). The baseline creatinine was significantly higher in documented AKI patients (0.88 vs. 0.73 mg/dl, p < 0.001). Similarly, the peak achieved creatinine was higher in those with documented AKI (2.87 vs. 1.97 mg/dl, p < 0.001). A secular trend in AKI documentation was also noted, with an increase in documentation rate of 2.5% for each year of the study (documentation rate 29% in 2004 to 49% in 2010, p < 0.001). 

Multiply-adjusted analyses of associations with formal AKI documentation appear in Table 2. The strongest association with documentation (by degree of statistical significance) was the baseline creatinine concentration (whereby patients with a higher baseline creatinine concentration were much more likely to have documentation of AKI than those with lower baseline creatinine concentrations). Other strong associations with AKI documentation included a higher peak creatinine and a higher achieved SOFA score. Surgical patients and black patients were much less likely to have formally documented AKI. The multivariable model discriminated well between patients who would and would not have AKI documentation with area under the receiver-operator characteristic curve 0.79 (0.78 – 0.81). 

### Relationship between formal AKI documentation and 30-day mortality 

In unadjusted analysis, formal documentation of AKI was associated with greater 30-day mortality – OR 1.65 (1.45 – 1.87, p < 0.001). Table 3 reports odds ratios for 30-day mortality based upon AKI documentation in unadjusted and sequentially adjusted forms. Although formal documentation of AKI was associated with death in unadjusted analyses, after adjustment for markers of severity of renal dysfunction and other illness, formal documentation of AKI appears to be protective with OR 0.81 (0.68 – 0.96, p = 0.02). In fact, after only adjusting for medical vs. surgical status and peak SOFA score, the OR for 30-day mortality in the documented vs. non-documented group was 0.82 (0.70 – 0.95, p = 0.009). 

We performed a series of interaction analyses to examine factors that would modify the association of AKI documentation and mortality. There was no evident effect modification across tertiles of baseline creatinine (from lowest to highest, adjusted ORs 0.83, 0.72, 0.85, respectively, p = 0.65 for interaction).There was an indication that AKI documentation was more strongly associated with decreased mortality among patients who achieved a higher serum creatinine concentration. By tertile of peak creatinine, the OR for documentation on 30-day mortality among those with peak creatinine ≤ 1.63 mg/dl was 0.95 (0.63 – 1.41); for those with peak creatinine 1.64 – 2.42 mg/dl the OR was 0.92 (0.70 – 1.23); and for those with peak creatinine ≥ 2.43 mg/dl the OR was 0.66 (0.50 – 0.86); p for interaction = 0.005. There was no detectable difference in the benefit of AKI documentation in surgical vs. medical patients (p for interaction = 0.63). 

### Interventions associated with formal AKI documentation 

Patients with documented AKI were much more likely to have a renal consult during admission (31% vs. 6%, p < 0.001). This effect persisted after multivariable adjustment for demographics, creatinine metrics, SOFA score, surgical and ICU status. In univariable analysis, the presence of a renal consult was not associated with 30-day mortality – OR 1.06 (0.90 – 1.27, p = 0.79). After adjustment for peak SOFA score, the presence of a renal consult was protective – OR 0.68 (0.56 – 0.82, p < 0.001). 

**Figure 2.**
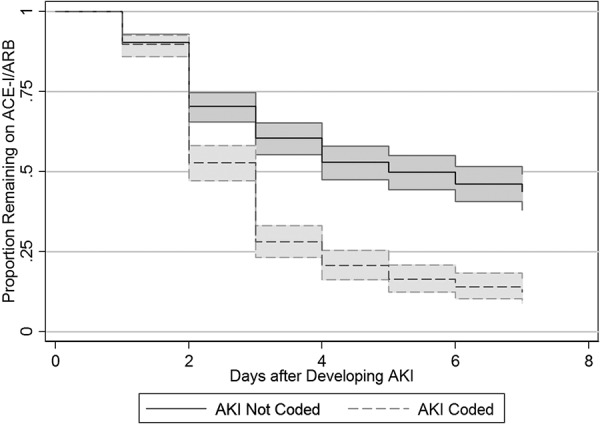
Kaplan-Meier survival curves illustrating the rate of ACE/ARB cessation among 710 patients admitted on an ACE or ARB who continued taking the drug until at least the onset of AKI. Time 0 represents AKI onset. Log-rank p < 0.001. ACE = angiotensin converting enzyme. ARB = angiotensin receptor blocker.

Patients with documented AKI were more likely to receive fluid boluses within 7 days of AKI, 64% vs. 45% in non-documented patients (p < 0.001). This effect remained after multivariable adjustment. Among patients who were receiving an ACE-inhibitor or ARB on admission, and throughout the hospital course to at least the day prior to onset of AKI (n = 710), AKI coding was strongly associated with rate of discontinuation of these drugs in univariable analysis (HR 2.04, CI 1.69 –  2.46, p < 0.001) and after adjustment for severity of renal disease, creatinine metrics, comorbidities, the presence of a renal consult, and medical vs. surgical service (HR 1.44, CI 1.16 –  1.78, p = 0.001) (Figure 2). 

### Validation of the use of ICD-9 billing codes as a proxy for AKI recognition 

A random subset (n = 194) of patient charts were reviewed to validate the use of ICD-9 billing codes as a proxy for AKI recognition by providers. Of these, 99 (51%) had formal AKI documentation defined by the assignment of ICD-9 billing codes for AKI. The two independent raters agreed on whether AKI was recognized or not in 184/194 (94.9%) patients, (κ-statistic 0.87, p < 0.001). AKI was recognized in 145 (74.7%) of patients within 7 days of AKI onset. Of these, 57 (40%) had evidence of recognition the day the patient met AKIN Stage 1 criteria, while 51 (35%) had evidence of AKI recognition the following day. Three (2%) patients had evidence of recognition the day prior to the patient meeting AKIN criteria. The presence of an ICD-9 billing code for AKI had a positive predictive value of 95% (89 – 98%) for AKI recognition in the medical record. The absence of a billing code was not a useful indicator of the absence of AKI recognition with negative predictive value 48% (38 – 58%). In this small sub-cohort, there was no statistical difference in 30-day mortality between those with recognized AKI and unrecognized AKI p-adjusted OR for recognition 0.68 (0.18 – 2.60, p = 0.58). 

### Sensitivity analysis 

To determine if recognized AKI was captured under other ICD-9 codes, we added a variety of codes to the definition of “formal documentation” (Supplementary Table 1). Of the 207 patients in our cohort who had one of these codes in their medical record, 71 had no other code for AKI. The addition of these codes did not alter the relationship between AKI coding and mortality. 

## Discussion 

Recent guidelines have standardized the approach to the diagnosis and treatment of AKI [5, 7]. But these guidelines cannot be adequately implemented in the absence of appropriate recognition of AKI. Beyond that, a provider who recognized AKI must convey that information effectively to the rest of the medical team – a process that depends in part on accurate progress notes. Within a severe AKI cohort, we utilized billing codes to identify patients in whom AKI was formally documented in order to determine risk factors for under-documentation of AKI and the association of documentation of AKI with mortality. 

In the overall cohort, only 43% of patients, all of whom had AKI as defined by a doubling in serum creatinine, had documentation in the medical record sufficient to subsequently lead to generation of an AKI billing code. This is higher than prior cohorts [8], but, given the fact that all subjects in our study experienced a doubling of serum creatinine, this represents striking under-documentation. 

Our sub-cohort, in which direct chart review was used to assess AKI recognition, suggests that many patients lacking formal documentation still had some evidence of provider recognition of AKI. Nevertheless, the association between documentation, subsequent therapeutic interventions, and mortality was strong. Failure of billing codes to capture all recognition of AKI would tend to bias our primary results towards the null hypothesis, as “recognized” AKI patients would be included in the non-documented group. Finding a benefit of documentation may be due to one of two factors. First, that the potency of the effect of recognition on clinical outcomes is enough to overcome the inherent biasing towards the null that comes with misclassification of this exposure via billing codes. Secondly, there may be a benefit to formal documentation itself that exceeds that obtained through less formal language. This could be due to increased communication among care providers, for example. We suggest that efforts to improve both AKI recognition and documentation should be formally evaluated. 

With our analysis of ACE/ARB discontinuation, fluid boluses, and nephrology consultations, we demonstrated that practice patterns differ between patients with documented and undocumented AKI, but can make no comment about a particular intervention that may have been most helpful. The fact that renal consult improved mortality after adjustment for peak SOFA score was intriguing, particularly given the fact that this was a non-dialyzed population, suggesting that renal consultation conferred its benefit via means other than dialysis. But it may be that the lack of an intervention (such as contrast or other nephrotoxin administration) may be equally important in the management of AKI. Indeed, fluid blousing (which occurred more often in the documented group) may be harmful in the setting of AKI [19, 20, 21]. Careful prospective studies may help to elucidate which practice patterns are particularly beneficial in this population. 

Our multivariable model demonstrates factors associated with AKI documentation. Higher baseline serum creatinine was strongly associated with AKI documentation, despite the fact that this cohort excluded patients with chronic kidney disease. This is likely due to the fact that, in patients with low baseline creatinine, a doubling of creatinine can take place without a dramatic absolute increase in serum creatinine. For example, a clinician may notice a change from 1.0 to 2.0 mg/dl more readily than a change from 0.5 to 1.0 mg/dl, despite the fact that both of these changes reflect the same (50%) reduction in glomerular filtration rate. Patients with low baseline creatinine may be an appropriate group to target for interventions designed to increase AKI recognition. 

Strengths of this study include its large size, availability of rich patient-level data throughout the hospital stay, and chart review-level comparison of formal vs. informal AKI documentation. The results of this study should be interpreted in the light of several limitations. First, there is likely significant residual confounding in this observational cohort, even after multivariable adjustment. Patients with greater severity of illness (not captured in our database) may be more likely to have AKI documented due to the greater clinical attention they receive, biasing our results against a benefit of AKI documentation (as seen in unadjusted analysis). Thus, the true beneficial effect of AKI recognition may be greater than that seen in our multivariable models. Second, the cohort contains only patients with relatively normal baseline renal function and who suffer a severe renal insult; it is unclear if these findings would extend to those with chronic kidney disease or to those with more mild AKI – though rates of documentation would likely be low in the latter group. Third, we cannot be certain when during the admission documentation occurred, limiting our ability to determine the temporal relationships between clinical covariates and documentation. Fourth, the etiology of renal failure is not known in all patients. Though the impact of documentation was similar between medical and surgical patients, it is unclear whether certain etiologies of AKI would benefit more from documentation. Fifth, we identified patients based upon serum creatinine concentration – not urine output – the failure to include patients with AKI based on oliguria alone may have impacted documentation rates. Finally, this cohort was assembled from a single health system; results may not be generalizable to study populations outside of the health system in which it was performed. 

In conclusion, approximately one half of patients in our study did not receive AKI documentation as evidenced by billing codes, and one quarter had no evidence of provider recognition by any indication using physician documentation. ICD-9 coding of AKI is a specific but insensitive surrogate of severe AKI recognition. The finding that AKI coding is associated with improved outcomes after adjustment for illness severity raises the possibility that better AKI recognition may improve such outcomes. Studies measuring the impact of efforts to improve AKI recognition are warranted. 

## Acknowledgments 

We would like to thank the Yuliya Borovskiy and the Penn Data Store for their assistance in assembling this cohort. We thank Craig Keane, Sherine Koshy, and Dina Amin for their assistance with obtaining pertinent coding data. This study was funded by NIDDK grant 1F32DK093223 awarded to FPW. 

## Conflicts of interest 

None. 

**Supplementary Table 1.** Billing codes for acute kidney injury.

ICD-9 Code	Description
Codes used in primary analysis	
584.5	Acute kidney failure with lesion of tubular necrosis
584.6	Acute kidney failure with lesion of renal cortical necrosis
584.7	Acute kidney failure with lesion of renal medullar (papillary) necrosis
584.8	Acute kidney failure with pathological lesion in kidney
584.9	Acute kidney failure, unspecified
Codes used in sensitivity analysis (in addition to above)	
580 (and subcodes)	Acute glomerulonephritis
581 (and subcodes)	Nephrotic syndrome
583 (and subcodes)	Nephritis and nephropathy, not specified as acute or chronic
586	Renal failure, unspecified
997.5	Urinary complications, not otherwise classified

**Table 1.** Patient characteristics and univariable comparison between patients with and without documentation of AKI. Continuous variables are expressed as mean ± standard deviation or median (IQR). Categorical variables are expressed as percentages.

	AKI not documented (n = 3,192)	AKI documented (n = 2,927)	Total (n = 6,119)	p-value (documented vs. not documented)
Demographics				
Male	47.7	57.8	52.1	< 0.001
Age, years	61.2 ± 16.7	61 ± 15.9	61.1 ± 16.4	0.64
Race				
Black	30.1	27.7	29	0.05
White	53.7	56.8	55	
Other	16.2	15.5	15.9	
Service				
Surgical	47.9	41	44.9	< 0.001
Medical	52.1	59	55.1	
Hospital course				
Length of Stay, days	12 (7 – 21)	17 (10 – 29)	14 (8 – 24)	< 0.001
Length of stay prior to AKI onset, days	3 (1 – 7)	4 (2 – 9)	3 (2 – 8)	< 0.001
Duration of AKI, days	6 (3 – 11)	9 (5 – 16)	7 (4 – 13)	< 0.001
ICU stay, days	2 (0 – 6)	4 (0 – 10)	2.5 (0 – 8)	< 0.001
Ever in an ICU	64.4	73.9	68.5	< 0.001
Ever ventilated	37.7	47.1	41.7	< 0.001
Comorbidities				
HIV	2.5	2.4	2.5	0.82
Malignancy	10.4	13	11.5	0.003
Congestive heart failure	31.7	39.5	35	< 0.001
Cardiovascular disease	10	9.6	9.8	0.65
Dementia	1.2	0.9	1.1	0.26
Diabetes mellitus	26.2	28.8	27.3	0.04
Hemiplegia	2.3	2.8	2.5	0.30
Metastatic solid tumor	9.2	10	9.5	0.31
Myocardial infarction	13.6	14.2	13.8	0.50
Liver disease	11.3	19.7	14.9	< 0.001
Pulmonary disease	16.6	13.8	15.4	0.004
Peripheral vascular disease	13.4	13.6	13.5	0.81
Rheumatic disease	3.3	3.3	3.3	0.943
Peptic ulcer disease	1.7	2.6	2.1	0.02
Creatinine kinetics				
Baseline creatinine, mg/dl	0.73 ± 0.26	0.88 ± 0.24	0.79 ± 0.26	< 0.001
Time from onset to doubling, days*	1 (0 – 2)	1 (0 – 2)	1 (0 – 2)	< 0.001
Peak creatinine, mg/dl	1.97 ± 0.93	2.87 ± 1.17	2.36 ± 1.13	< 0.001
Nadir-to-Peak creatinine, mg/dl	1.25 ± 0.77	1.99 ± 1.11	1.57 ± 1	< 0.001
Other labs				
Nadir bicarbonate, meq/l	18.9 ± 5	17.3 ± 4.9	18.2 ± 5	< 0.001
Peak potassium, meq/l	5.2 ± 0.9	5.4 ± 0.9	5.3 ± 0.9	< 0.001
Peak BUN, mg/dl	42 ± 26	61 ± 35	50 ± 31	< 0.001
Peak WBC count, thousands/ul	19.3 ± 15.3	22.3 ± 22.2	20.6 ± 18.6	< 0.001
Nadir hemoglobin, mg/dl	8.3 ± 2	7.8 ± 1.8	8.1 ± 1.9	< 0.001
Nadir sodium, meq/l	132.1 ± 4.7	131.3 ± 4.8	131.8 ± 4.8	< 0.001
Acuity of illness				
Peak SOFA score	5.1 ± 3.5	7.1 ± 3.9	6 ± 3.8	< 0.001

*Patients with documentation had longer time from start of AKI to doubling of creatinine, though not reflected in the median (IQR). ICU = intensive care unit; HIV = human immunodeficiency virus infection; BUN = blood urea nitrogen; WBC = white blood cell; SOFA = sequential organ failure assessment.

**Table 2.** Multivariable adjusted odds ratios (95% confidence interval) for documentation of AKI during the hospital course according to patient characteristics. Peak and nadir lab values reflect those that occurred during the AKI episode.

	Odds ratio for documentation (95% CI)	p-value
Demographics		
Male	0.79 (0.69 – 0.91)	0.001
Race		
Black	0.81 (0.70 – 0.94)	0.004
Service		
Surgical	0.64 (0.56 – 0.73)	< 0.001
Hospital course		
Length of stay, per day	1.01 (1.00 – 1.02)	0.003
Length of stay prior to AKI onset, per day	1.00 (0.99 – 1.01)	0.83
Duration of AKI, per day	1.03 (1.02 – 1.04)	< 0.001
ICU stay, per day	0.99 (0.98 – 1.00)	0.03
Comorbidities		
Diabetes mellitus	1.18 (1.02 – 1.35)	0.02
Malignancy	1.30 (1.07 – 1.58)	0.008
Congestive heart failure	0.98 (0.85 – 1.13)	0.77
Liver disease	1.24 (1.03 – 1.49)	0.03
Peptic ulcer disease	1.21 (0.79 – 1.85)	0.39
Pulmonary disease	0.75 (0.63 – 0.90)	0.002
Creatinine kinetics		
Baseline creatinine, per mg/dl	4.44 (3.13 – 6.28)	< 0.001
Time from onset to doubling, per day	0.94 (0.91 – 0.96)	< 0.001
Peak creatinine, per mg/dl	1.89 (1.72 – 2.09)	< 0.001
Other labs		
Nadir bicarbonate, per meq/l	0.98 (0.97 – 1.00)	0.04
Peak potassium, per meq/l	0.88 (0.81 – 0.96)	0.002
Peak BUN, per 10 mg/dl	1.03 (1.00 – 1.06)	0.03
Peak WBC Count, per thousand/ul	1.00 (1.00 – 1.00)	0.47
Nadir hemoglobin, per mg/dl	0.98 (0.94 – 1.02)	0.38
Nadir sodium, per meq/l	0.98 (0.97 – 1.00)	0.01
Acuity of illness		
Peak SOFA score, per point	1.06 (1.03 – 1.08)	< 0.001
Year of admission		
Years after 2004, per year	1.20 (1.17 – 1.25)	< 0.001

ICU = intensive care unit; BUN = blood urea nitrogen; WBC = white blood cell; SOFA = sequential organ failure assessment.

**Table 3.** Sequentially adjusted models examining the impact of documentation on 30-day mortality.

Impact of Documentation on 30-day mortality	OR	p
Unadjusted	1.65 (1.45 – 1.87)	< 0.001
Above + age, gender, race, year of admission	1.69 (1.48 – 1.92)	< 0.001
Above + surgical + ICU	1.44 (1.26 – 1.65)	< 0.001
Above + malignancy, CHF, diabetes mellitus, liver disease, pulmonary disease	1.35 (1.17 – 1.55)	< 0.001
Above + baseline creatinine, peak creatinine, time to doubling of creatinine, nadir bicarbonate, peak BUN, peak potassium, nadir sodium	0.94 (0.80 – 1.11)	0.45
Above + peak SOFA Score	0.81 (0.68 – 0.96)	0.02

Logistic regression examining the association of AKI Documentation on 30-day Mortality. Sequential adjustment evidences protective effect of AKI documentation. Final model includes all covariates identified as significant in unadjusted models of mortality. ICU = intensive care unit; SOFA = sequential organ failure assessment; CHF = congestive heart failure; MI = myocardial infarction.
